# Does the current state of biomarker discovery in autism reflect the limits of reductionism in precision medicine? Suggestions for an integrative approach that considers dynamic mechanisms between brain, body, and the social environment

**DOI:** 10.3389/fpsyt.2023.1085445

**Published:** 2023-02-22

**Authors:** Eva Loth

**Affiliations:** Department of Forensic and Neurodevelopmental Sciences, Institute of Psychiatry, Psychology and Neuroscience, King’s College London, London, United Kingdom

**Keywords:** autism, biomarker, precision medicine, neurodiversity, systems biology, reductionism, neurodevelopmental conditions

## Abstract

Over the past decade, precision medicine has become one of the most influential approaches in biomedical research to improve early detection, diagnosis, and prognosis of clinical conditions and develop mechanism-based therapies tailored to individual characteristics using biomarkers. This perspective article first reviews the origins and concept of precision medicine approaches to autism and summarises recent findings from the first “generation” of biomarker studies. Multi-disciplinary research initiatives created substantially larger, comprehensively characterised cohorts, shifted the focus from group-comparisons to individual variability and subgroups, increased methodological rigour and advanced analytic innovations. However, although several candidate markers with probabilistic value have been identified, separate efforts to divide autism by molecular, brain structural/functional or cognitive markers have not identified a validated diagnostic subgroup. Conversely, studies of specific monogenic subgroups revealed substantial variability in biology and behaviour. The second part discusses both conceptual and methodological factors in these findings. It is argued that the predominant reductionist approach, which seeks to parse complex issues into simpler, more tractable units, let us to neglect the interactions between brain and body, and divorce individuals from their social environment. The third part draws on insights from systems biology, developmental psychology and neurodiversity approaches to outline an integrative approach that considers the dynamic interaction between biological (brain, body) and social mechanisms (stress, stigma) to understanding the origins of autistic features in particular conditions and contexts. This requires 1) closer collaboration with autistic people to increase face validity of concepts and methodologies; (2) development of measures/technologies that enable repeat assessment of social and biological factors in different (naturalistic) conditions and contexts, (3) new analytic methods to study (simulate) these interactions (including emergent properties), and (4) cross-condition designs to understand which mechanisms are transdiagnostic or specific for particular autistic sub-populations. Tailored support may entail both creating more favourable conditions in the social environment and interventions for some autistic people to increase well-being.

## Introduction

Over the last 40 years, much of what (we thought) we knew about autism has changed or has been modified; ranging from the prevalence of autism to the conceptualisation and definition of autism, through to the research goals, priorities and conduct of research.

Autism was once considered a rare condition, with prevalence estimates of 3–4 in 10,000 individuals in the 1970s (1). It was also commonly considered a “severe disorder.” The *qualitative* differences in the clinical presentation were highlighted, such that some authors argued it would be almost impossible for a non-autistic person to imagine what it is like to be autistic (2).

By contrast, currently 1–2% of the population or approximately 78 million people worldwide are estimated to be autistic—which represents a 20 to 30-fold increased prevalence (3, 4). One likely factor in this increase are various changes in the definition and diagnostic criteria over time. In the two major diagnostic manuals, the Diagnostic and Statistical Manual (DSM) and the International Classification of Diseases (ICD), autism has always been defined based on a set of behavioural features (or symptoms) rather than aetiology or biological characteristics [DSM-5 (5)]. However, across the latest revisions, the notion of *qualitative* differences in core domains has given way to the view of autism as a spectrum, with *quantitative* differences in autistic traits and a “broader autism phenotype” (6) shading into so-called “normality.” Arguably, these changes have led to a decrease in specificity (7, 8) and an increase in the proportion of people diagnosed with autism without intellectual disability (ID) (from 31% in the 1980 to 61–83%) (4, 9). Moreover, from an ontological perspective, the neurodiversity paradigm, informed by first-person experiences, has criticised the ICD-DSM definitions of what is autism and instigated a fundamental shift from deficit models to emphasising differences in autistic perception, cognition and experiences (10–12). Also, while autism has originally been a male-dominant condition, recent studies indicate that differences in the behavioural presentation in females might mean that the actual sex ratio is less pronounced than originally thought (13, 14). Furthermore, co-occurrence of other neurodevelopmental, neuropsychiatric, and medical conditions has been noted. Whereas up until the DSM-5 (15) diagnosis of autism and ADHD was mutually exclusive, newer reports indicate that between 28 and 53% of autistic children meet criteria for ADHD and between 7 and 37% criteria for an oppositional defiant disorder or conduct disorder (16, 17). 42% of autistic adults—notably females diagnosed in adulthood (18)—have a lifetime prevalence of anxiety disorder and 37% of depressive disorder (19). Around 4–8% of autistic people have epilepsy, which increases to 20–40% in autistic people with Intellectual Disability (20). Autism also involves a markedly higher premature mortality rate compared to the general population owing to numerous mental health and medical conditions, notably a 9-fold increase in suicide rate and 40-times increased mortality rate from epilepsy (21). There is also increasing awareness that multiple systems of the body are affected, which include—alongside the neurological system—metabolic, gastrointestinal, immunological, and mitochondrial systems (22), and connective tissue (23), though it remains unclear to what extent they may play an etiological role.

This change in the autistic population has also affected changes in research priorities.

Although heterogeneity has been known for a long time (24), a dominant research goal was to develop a *unifying* theory that explains *all* symptoms in *all* autistic people (25). This has given way to a view that multiple cognitive or biological characteristics may underpin different clinical features (26). Indeed, no cognitive or biological characteristic has been identified that characterises all or most autistic people. We recently showed that across the most influential areas of autism research, small (*d* = 0.21) to large effect sizes (*d* = 1.1) in cognitive, EEG, and MRI studies translate to 45 to 63% of autistic people falling *within* 1 Standard Deviation of the typically developing control group; i.e., they do *not* have an atypicality in a statistical sense (27). Rødgaard et al. (28) showed that effect sizes in these areas decreased by up to 80% over the past 20 years, presumably owing at least in part to the increased heterogeneity of study participants.

As a consequence, many researchers have been sceptical that such a unifying biological characteristic or “final common pathway” exists among this diverse group. Some believe that it is important to understand this heterogeneity and shifted the goal to identifying biological “subgroups” to make more accurate clinical predictions (see below). Others take lacking evidence of a shared biological basis to argue for abandoning the categorical diagnosis of autism (29, 30) and indeed neurodevelopmental/neuropsychiatric conditions altogether (31), pointing to at times detrimental repercussions for clinical pathways and care. A third view is that the high prevalence rates reflect an inflation of autism diagnosis in people with broader atypicalities in the areas of social communication and repetitive interests. In particular, Mottron (7) proposed a research strategy that returns to a more narrow definition of autism, termed “prototypical autism,” to identify the biological basis of people with a more homogeneous, qualitatively recognisable clinical presentation, notably in early development. However, shared among these different views is the recognition that the more diverse is a diagnostic group, the harder it is to make meaningful, clinically relevant predictions about an individual from the group information (32).

In this contribution to the special issue on the question “Is autism a biological entity?,” I will first review the origins and concept of precision medicine approaches to autism, and summarise recent findings from the first “generation” of biomarker studies to identify and characterise biological subgroups. The second part turns to discussing some methodological and conceptual challenges in this research agenda; in particular potential limitations of the reductionist approach in biomedical science, which tends to parse complex issues into simpler, more tractable units. The third part draws on insights and examples from systems biology, developmental psychology and neurodiversity to outline an integrative approach that considers the dynamic between biological and social mechanisms to understanding the origins of autistic features in particular conditions and contexts.

## Precision medicine approaches to autism

Precision medicine approaches to autism were motivated by the recognition that a categorical, symptom-based diagnosis of autism itself does not enable us to make accurate predictions about a particular autistic person, such as their likely natural development, treatment/support needs as well as efficacy of specific therapies, or the underlying cause of the condition (33–36). This approach, as much as the term *precision medicine* itself, follows a trend that first started in internal medicine and that was then imported to psychiatry; reminding us that heterogeneity is not only a phenomenon specific to autism but in fact prevalent across medical and psychiatric conditions. It aims to match new mechanism-based treatments with objective tests (predictive biomarker) to estimate which therapy is most beneficial for this particular person (37). Hence, a key tenet is that interventions/support will be more effective if they target underlying mechanisms rather than treating symptoms (i.e., symptomatic treatment) and that mechanisms—and thus treatment responses—may differ even between people with the same umbrella diagnosis (38). It also stresses that early identification and intervention closer to causal mechanisms likely have the strongest lasting benefits on cognitive, social, and emotional development because of substantial underlying brain growth and plasticity over the first months and years of life. This opens the possibility to shift the trajectory toward growth of strengths as opposed to amelioration of symptoms.

To enable this approach, a key pillar of precision medicine is identification of “biomarkers.” The original definition by the Biomarker Working Group, (39) stressed a biomarker as a biological characteristic that can be *objectively* measured (as opposed to clinical judgement that is somewhat subjective). The Biomarker, EndpointS and other Tools (BEST) Resource of the FDA-NIH Biomarker Working Group (40) divided biomarker types by their specific clinical purpose (“contexts of use”). These include to aid in (1) the early detection of a condition, possible before behavioural features arise (likelihood biomarker), (2) more objective and reliable diagnosis (diagnostic biomarker), (3) predicting the “natural” developmental course without any intervention (prognostic biomarker), and (4) predicting treatment benefit as well as potential side effects (predictive biomarker), or for other purposes. A biomarker could be any measurable characteristic, from a gene to molecular marker, brain structural or functional read-out, cognitive or behavioural tests. Note that in homogeneous conditions, a biomarker should apply to all or most people with that condition—corresponding with the search for universal and specific characteristic(s) of autism discussed above. A biomarker may also apply to most/all individuals in a situation where different causes give rise to a “final common pathway” at one intermediate level but additional factors influence behavioural/clinical outcomes (41). By contrast, for heterogeneous conditions without a final common pathway, each of these biomarker types are variants of a *stratification biomarker* and only apply to a particular sub-group (see below for the interpretation of this term by the non-scientific autistic community). For example, it may help to objectively diagnose a specific subgroup of autistic people; such as those with increased likelihood for late onset epilepsy. A biomarker could be categorical (e.g., presence/absence of a gene), a quantitative measure that designates biomarker ‘positivity’ from a certain cut-off point, or it could be a panel comprising different measures.

If a diagnostic biomarker was found, it would redefine autism as a “biological entity.” If it were found for a sub-population, it would make a subpopulation a “biological entity.” This is effectively the case for several monogenic conditions that involve strong likelihood (penetrance) for autism. For example, approximately 0.5–1% of autistic people have Phelan McDermid Syndrome (PMS), and conversely 70–80% of people with PMS meet criteria for autism (42). Other genes are more pleiotropic, leading to a range of neurodevelopmental/psychiatric conditions (e.g., Fragile X Syndrome, 22q11.2). By contrast, a transdiagnostic biomarker is a biological characteristic or state indicative of a clinical feature that is shared across people with different conditions, such as neuroendocrine and neuroinflammatory markers of stress-related depression (43).

Thus, a biomarker is a biological characteristic or state *at a certain moment in time*. It does not necessarily have to be stable across development; i.e., it could be transient, and only detectable say in early development, and—as argued below- may vary across contexts or conditions. It is also not necessarily caused by a gene—but could result from environmental factors, for example early trauma, deprivation, or stress etc. In this regard, a biomarker is different to an *endophenotype*, which is thought to be relatively stable and must be inherited (44). Many biological processes in the brain, such as (increased) myelination, synaptic (over)production, synaptic pruning (which all play a part in cortical thickness) are *experience-dependent* biological processes, and therefore affected by exogenous as well as endogenous events.

Hence, it could be the individual child, the environment and/or the interaction between individual and environment (individual’s life experience) that impacts biological developmental processes. It is this effort to identify biomarkers for autism that has substantially changed methodologies and the research culture over the past years.

### Biomarker studies and subtyping approaches in EU-AIMS and AIMS-2-TRIALS

This approach is exemplified by EU-AIMS and AIMS-2-TRIALS, which are two linked consortia that were specifically set up to identify biomarkers in autism (45, 46). In EU-AIMS (2012-2019), the first generation of biomarker studies comprised two complementary approaches: (1) large-scale cohort studies to parse heterogeneity and (2) gene-first approaches to identify mechanisms in *a priori* genetically-defined subgroups.

First, to get the statistical power to recruit and assess larger cohorts needed to subdivide heterogeneous idiopathic autism groups, we had to shift from small-scale studies (typically including 15–30 participants per group) to multi-centre studies. The *Longitudinal European Autism Project* [LEAP, (47, 48)] uses a case-control accelerated longitudinal design (*N* = 420 autistic, 350 non-autistic) to identify subgroups within the autism group. The categorical autism diagnosis is needed as a reference point. *Accelerated longitudinal* means that four cohorts of children, adolescents, adults without intellectual disability and adolescents/adults with mild intellectual disability were simultaneously recruited and then followed up on two time-points within 8 years. Deliberately there were few participant exclusion criteria. We allowed all co-occurring medical and mental health conditions (except psychosis) at a time where many studies excluded participants with co-occurring ADHD and included people with mild intellectual disability (ID) (around ∼18%) when most neuroimaging studies excluded people with ID. The sample was deliberately “enriched” for females (with a 1 female to 3 male ratio) to conduct sex-stratified analyses at a time when many studies focused on males only. The age range was chosen because brain imaging was a core assessment and we were not confident about viability of preschool MRI scanning at the time. Whereas most previous studies assessed participants on one or a few measures to test a specific hypothesis, each participant is comprehensively assessed across multiple domains and “levels” (“deep-phenotyped”) to test/compare some of the most established hypotheses (theory of mind, executive functions, social motivation) and emerging hypotheses at the time (e.g., excitatory/inhibitory imbalance). More exploratorily, we aimed to link different assessments to map differences in genes to downstream molecular, brain systems level, cognitive and behavioural features. For the first time in autism research, we obtained qualification advice from the European Medicines Agency (EMA) to increase the chances that data generated by the study would be accepted for biomarker qualification for particular “contexts of use” (47).

Our analysis strategy comprised distinct steps. First, we conducted mean-group comparisons and dimensional analyses for each measure. Significant mean-group differences were found in functional connectivity [(49), as indexed by degree of centrality but not using Independent Component Analysis, (50)], social attention patterns, including temporal profiles (51), biomotion (52), theory of mind, emotion recognition (53) early-stage face processing [N170 latency, (54)] and functional activation during reward processing (55). No significant mean group differences were observed in functional activation in brain regions implicated in theory of mind (56), emotion recognition, EEG power spectrum or functional connectivity (57), and (largely) brain anatomy (58).

However, for biomarker discovery mean group differences should be treated as a starting point only. As stated above, the difference between a statistically significant and non-significant group comparison could be a matter of 45 vs 55% of autistic people performing below say 1 Standard Deviation (SD) of the “typical mean” (27); so it may be more indicative of the size of a potential subgroup. Therefore, we moved our focus from mean-group comparisons to identify individual profiles; and subgrouping approaches.

On the one hand, we defined subgroups *a priori* by sex/gender, age/developmental stage and other variables putatively affecting sub-populations and examined differences in neurobiology. This revealed similar effects of sex and diagnosis, as well as some sex-by-diagnosis interactions in intrinsic brain function (59). Also both autistic and non-autistic females showed on average stronger social attention than autistic and non-autistic males when watching static images, with subtle differences in dynamic looking patterns over time (51). We also carried out *sensitivity analyses* to examine potential differences between autistic participants who meet vs. do not meet ADOS/ADI cut-off scores. On the whole, sensitivity analyses increased somewhat but not drastically effect sizes, but in some instances crossed the significance level (p-value) divide (e.g., on some theory of mind tests). However, whereas these analyses predominantly reflect differences in the strengths of social-communicative features or repetitive behaviours, it remains to be tested whether commonalities in cognitive or biological characteristics may be more likely captured by clinical ‘prototypicality’ (7).

Secondly, we aimed to make individual predictions based on normed scores of cognitive or brain development using growth charts and then used data-driven approaches to identify subgroups. Reference scores or growth charts are routinely used in paediatrics to interpret a child’s weight/height, or in IQ or educational assessments using standardised scores. More recently, such growth charts have also been created for brain development (60–62) and function (54) to assess individual variability relative to expectations based on a person’s age, sex or other variables. We can then use these scores in clustering or other multi-variate analyses to identify subgroups at the clinical, cognitive level, neurobiological level, or a combination thererof.

This approach identified diverse atypicalities in brain anatomy in the autism group, which were not located in the same regions in all autistic participants and would have gone undetected in mean-group comparisons of *a priori* regions of interest (58, 63). For example, autistic participants showed highly individualised patterns of both extreme right- and leftward lateralisation, particularly in language, motor, and visuospatial regions. Language delay explained most variance in extreme rightward patterns whereas strengths of autism core features explained most variance in extreme leftward patterns (64). We also identified cognitive subgroups using robust clustering based on behavioural expression recognition performance across three tests. These subgroups were related both to clinical features (explaining more variance in social adaptive function than subgrouping by IQ) and functional activation in amygdala activation (53).

The question is then whether these subgroups can be used to inform prognosis or treatment choices. This approach is exemplified by the way speed of early-stage face processing (N170 latency, as measured by EEG) was investigated as prognostic biomarker (54, 63). Face processing has long been suggested as an early marker of atypical social information processing in autism (66). Here, we first replicated significant mean-group differences with medium effect size. Although slower N170 responses was only found in a subset of autistic participants, this subgroup showed on average poorer social prognosis as measured by adaptive socialisation skills over an 18-month follow-up period. In addition, N170 latency was associated with lower fMRI BOLD responses to faces in the fusiform gyrus during an fMRI task and polygenic scores for autism, triangulating links to social biology. Moreover, simulations showed that a distributional data-driven cut-off used to define “N170 latency biomarker positivity” as enrichment marker predicted improvements of power in simulated clinical trials targeting social functioning. From an ethical perspective, it is important to know what developmental trajectory likely entails what kind of difficulties for participants to weigh up likely costs/benefits in taking part in a clinical trial. For the first time in autism research, the N170 has now been included in the biomarker work programme by the FDA [led by the ABC-CT consortium, (67)] and has been supported by the EMA as baseline covariate. The longitudinal character of LEAP (with the ongoing 3rd assessment wave) affords subgrouping performed based on clinical/functional development and to then examine markers that may relate to different developmental changes/trajectories (e.g., using social attention to predict if adaptive function stayed the same, improved, or decreased relative to age expectations).

In sum, biomarker approaches to autism, and the ambition of precision medicine to transform healthcare, has shifted the focus from mean-group comparisons to predictions about individuals. It led to larger-scale, comprehensively characterised cohorts, set new standards in methodological rigour, robustness, replicability and temporal stability (67, 68), and development and use of innovative advanced ‘features’ [(e.g., from ROIs to connectopics (69), from cortical thickness to cortical gyrification (70) areas of interest to temporal dynamics in eye-tracking (51)]. It also changed the research culture by instigating both multi-disciplinary and cross-consortia collaborations (46).

However, so far we have not found a clearly delineated biologically-defined autism subgroup. There remains considerable overlap between subgroups in terms of clinical features and separately assessed biological characteristics. Thus, the predictive value is probabilistic, in that biomarker positivity increases likelihood of a certain outcome. Although it is possible that the predictive value may be higher for smaller subgroups (say < 10%), which could be highly clinically relevant, there is a danger of trying to slice autism into ever smaller sub-groups just to find a “biological entity.”

Before discussing potential technological and conceptual factors in these findings, advances from the complementary approach that starts with a particular genetic “subgroup” are reviewed.

#### Gene-first approaches

Gene-first approaches focus on a particular neurogenetic or monogenic (sub-)group to identify mechanisms and markers linked to a specific gene or gene product in order to identify treatable molecular targets. Based on the premise that some genes may converge on common molecular pathways [e.g., affecting synapse development, (71)] the subsequent goal is then to explore whether any atypicality generalises to other ‘types’ of syndromic or even idiopathic autism. One example of this approach is biomarker research in Phelan McDermid Syndrome (PMS). PMS was originally defined as deletion of the distal long arm of chromosome 22 and is also called 22q13.3 deletion syndrome (72). Later it was identified that deletions or haploinsufficiency of SHANK3 cause many clinical features. However, the presentation and needs of autism in PMS substantially differ from that of many idiopathic autistic individuals, largely due to severe to profound ID in 75% of cases (42).

SHANK3 is a postsynaptic scaffolding protein at glutamatergic synapses involved in synapse development and function, and regulation of dendritic spine morphology. At the systems level, this predicts to result in perturbations of the Excitatory/Inhibitory balance, generating broad hypotheses of functional differences.

Biomarker studies using EEG found group-level atypicalities in brain functional integration and connectivity, which are likely also reflective of ID, and significantly increased alpha-gamma phase bias (73). However, findings of other ‘proxy markers’ of E-I imbalance such as reduced Mismatch Negativity, gamma band atypicalities, or 1/f are more mixed ((74), in press).

Also more variability among people with Phelan McDermid Syndrome at both the behavioural and molecular levels is now being reported than we first expected. In a recent collaboration with Mount Sinai we investigated differences in social (vs. non-social) orienting in 67 children with PMS, 45 autistic children and 28 TD children. Social orienting was previously hypothesised to be an early marker of social cognitive atypicalities in autism (75). While at the group level, children with PMS responded significantly less often to both stimulus types, some PMS children in fact did respond to both, others almost to none, and others selectively to either social or non-social stimuli ((76), in press). Likewise, molecular studies now show that people with specifically SHANK3 point mutation actually have variable expressions of SHANK levels that cannot solely be attributed to deletion size or location (77).

In sum, even when aetiology is known, it has turned out to be a long way to map the mechanistic pathophysiology from a particular gene to shared or variable biological, behavioural and clinical features. The next section discusses technological/methodological and conceptual factors that may have contributed to difficulties in finding markers and mechanisms that characterise autism subgroups.

## Methodological and conceptual factors in biomarker discovery

### Are our current methods and technologies not reliable enough to identify subgroups?

Evidently, the results we get depend on the technologies, methodologies and methods we use and the signal of each measure occurs in the context of noise and measurement error. Here, this illustrated using neuroimaging as an example as advances in neuroimaging have chiefly influenced neurodevelopmental research, but similar considerations may also apply to other technologies and methods. First, for a technology to be used as a clinical tool it is not trivial that acquisition rates and data quality can be very variable and are to some extent systematically related to participant characteristics (age, IQ, sensory sensitivities etc.). For example, MRI scanning is particularly difficult in preschoolers with neurodevelopmental conditions or people with ID. As a consequence, these sub-populations were often left out from neuroimaging studies. Hence, we need more tools to reliably acquire neuroimaging data in children, and people with complex needs (including silent sequences, motion correction procedures).

Some neuroimaging indices (e.g., voxels in Magnetic Resonance Spectroscopy) are still very coarse; and do not allow us to specify specific neuronal differences. This is exemplified by pre-clinical work showing low correspondence between atypicalities in particular neuronal signalling and neurotransmitter concentrations. Thus, it is likely that limited accuracy or granularity of some measurements contribute to moderate relationships between candidate biomarkers and clinical outcomes.

Recent studies also reported poor test-retest reliability of resting state functional connectivity, with average Intra Class Correlation (ICC) of.29 (78) and task activation with average ICC of.39 (79). These findings highlight a related issue in that test results may not only reflect limited measurement accuracy itself but the fact that the read-outs we obtain (e.g., functional connectivity in certain networks at “rest” or during task performance) are only a snapshot *at a particular moment in time* and *in a particular context*. For example, regional activation (e.g., fusiform gyrus) can vary substantially across different conditions or tasks in the same individual within the same scan session. Unless we know that one condition is most clinically relevant, and why (amygdala activation to happy vs. fearful faces, or collapsed), it may be unclear which feature to carry forward as *candidate biomarker* and use to link to clinical features.

Likewise, many other candidate biomarkers are sensitive to condition and context effects. For instance, serotonin levels are known to vary across different times of the day (80), microbiome varies as a function of diet (81) that is influenced both by environmental factors and personal preferences. Research on double-empathy (12) shows that the ability or accuracy in understanding another person’s perspective may depend on the relationship between self and other, such that even ‘reliable’ test scores on repeated experimental theory of mind tasks may still have poor face validity if they fail to capture the way someone interprets different real life social interactions. Hence, in contrast to the sometimes tacit assumption that candidate biomarkers measured in the laboratory at a certain moment in time should be representative for this individual’s true state at a given *developmental stage*, potential variations across contexts or conditions are often unknown or untested. Although in biomarker research, these moderating factors should be established as part of “pre-analytic validation,” the fact that they are often not considered may also reflect some implicit conceptual assumptions.

### Are biomarker approaches too reductionistic?

Reductionism has been the predominant paradigm in biomedical science since Descartes. The fundamental approach of methodological reductionism is to understand complex issues, such as systems or processes, by dividing them into simpler and more tractable constituent units and their interactions. Methodological reductionism has been—often successfully- applied to the diagnosis, treatment and prevention of medical conditions, such as tumor type in predicting treatment and progression (37, 82). Ontological reductionism (not necessarily embraced by all precision medicine approaches) makes the stronger assertion that “higher” levels can be explained by “lower” levels (e.g., social sciences by psychology, psychology by biology, biology by chemistry, chemistry by physics). In autism research, we tend to separately investigate immune markers, metabolomics, brain structure or brain function as candidate marker for particular outcomes. However, the focus on specific “parts” of an individual neglects (1) that the interaction between them can produce a whole that is bigger than the sum of its parts—emergent properties, (2) the context or condition in which particular characteristics or processes operate, while (3) the focus on individuals neglects interactions between the person and their social environment. In brief, when approached through a “reductionist lens,” personalised medicine may not only risk overlooking the person (83), but also divorces the person from their environment. Several separate traditions challenge the reductionist approach to precision medicine.

#### Systems biology: Integrating brain and body

Systems biology assumes that the whole cannot be understood by studying the individual constituent parts and explicitly appreciates holistic and dynamic characteristics of ‘systems’ during particular operations over time (84). One example used to support this argument is the human genome project, which shows that from a relatively small number of 20,000 to 25,000 genes, one individual carries on average 3 million genetic variants, which interact to encode for nearly 100 trillion cells in the human body. This rich information is not only derived from the genes themselves and the interaction between genes, but also interactions with their gene products. Critically, between each hierarchical level (DNA to RNA, RNA to proteins) modifications are made, such that thousands of molecules interact with one another to give rise to a complex regulatory network and particular phenotypic characteristics.

A systems biology approach to precision medicine aims to take into account and integrate information from multiple sources, including genes and the environment, and different ‘parts’ of brain and body, to make predictions about an individual. The question is then how properties *emerge* from the addition and/or interactions of multiple components in particular conditions, and over time [see also (85)]. This may help us to understand how even a rare variant (e.g., SHANK3 point mutation) can lead to different clinical or behavioural presentations in different people depending on their genomic background (86), environmental and/or stochastic factors, or why identical twins can be discordant for autism or differ in their presentation of autistic features (87).

Considering the *context* or *condition* in which particular functions operate and develop also gives rise to questions, such as how brain and cognitive development are affected by acute and persistent stress (experienced endogenously or exogenously, [see example in the next section], atypicalities in sleep, or compromised gastrointestinal or immune functions (88). For example, the gut is linked to brain development and function via the parasympathetic nervous system, the immune system, the gut endocrine system and neuroactive metabolites and neurotransmitters directly produced in the gut (89). Some of these effects are likely bi-directional and dynamic over time, and these mechanisms may be missed when studying markers of brain and other internal systems separately.

#### Placing the individual in their social context

The next step is to bring the autistic person back into their social environment. Most cognitive and neurobiological studies of autism (regardless of whether they explicitly aim to identify biomarkers) tend to examine autistic participants on their own, with relatively little consideration of environmental and social factors on behaviour and development. Speculatively, some factors in this may be the historic image of the “autistic aloneness,” suggesting that autistic people were less influenced by their environment than non-autistic people, and recognition of high heritability, such that environmental factors were deemed less critical in searching for the causes of autism. Also rejection of the psychodynamic “refrigerator mother” hypothesis may have resulted in a tendency of the field to altogether shy away from social dynamics. In any event, the result has been that we often examine the autistic person in social isolation, which paradoxically includes studies of their *social* (cognitive) development. Insights from social psychology warn that a reductionist focus on an individual’s (or group’s) actions without acknowledging the dynamics of inter-actions and re-actions can readily lead one to pathologise the individual (90).

The importance of social mechanisms in development, behaviour and well-being has been the subject of several separate traditions in developmental psychology, social psychology, and psychiatry. With regards to autism, some of these arguments have been vividly brought to the fore by neurodiversity proponents (10, 12, 91). Some proponents have put forward a two-component definition of neurodevelopmental conditions. “Impairment as objective scientific component” (which acknowledges the brain basis, as indeed implicit in the term *Neuro*-diversity) and a “normative, socially negotiated component.” It is argued that a significant portion of distress and disablement—including anxiety, depression, suicidal ideation and suicide—is caused by social barriers and “ableist norms” created by a non-autistic sociality, rather than the cognitive traits associated with autism themselves (92). Thus, by locating the source of a great proportion of difficulties in the social environment (which includes the psychological and biomedical community itself) it suggests a so-called “downward causation” from the larger system to the individual.

Here, the hypothesised interplay between downward (social) and upward (biological) mechanisms is illustrated by stress reactions. It is well known that adverse social experiences (stress, abuse, trauma, neglect), notably during early development, substantially impact brain and social, cognitive and emotional development in *non-autistic people*, and significantly increase likelihood to develop mental health or behavioural issues (93, 94). While under normal conditions, acute stress responses, such as increased heart rate, surge in stress hormone levels, adrenalin rush etc, go back to baseline when the stressor is relieved, recurrent experiences of abuse or neglect result in constant activation of the stress system even at times when no apparent (physical) harm is present (95). A stress system that is permanently on high alert impacts the function of other developing systems. This generates predictions of the effect of stress on social and emotional development, and mental health, in autistic people. In fact, autistic people are more likely to experience social adversities, such as stigma or bullying than non-autistic people (96, 97). Moreover, it is likely that some core features of autism (sensitivity to sounds, difficulties adapting to unexpected changes) interact with environmental factors in creating more frequent and intense experiences of stress and trauma in (for neurotypicals) relatively mundane situations (e.g., eating lunch in a noisy kindergarten or canteen, going to the airport). Those intense stress reactions can drastically affect a person’s functioning both at a certain moment in time and across prolonged periods. For instance, they may create further anxiety due to uncertainty about when and how the next sensory overwhelming experience may happen in an unpredictable environment. Consequently, the effect of sensory sensitivity on stress may be mediated both by changes in hypersensitivity as well as changes in environmental conditions, such that a child hypersensitive to sounds may function better in an environment where occurrences of loud unexpected noises are reduced. The example highlights two points: First, it illustrates that the Research Domain Criteria (RDoc) approach of studying different domains (social, arousal etc.) as well as behavioural/clinical features separately might risk missing critical interactions in the functioning and development of these domains. Second, we cannot make a prognosis about an (autistic) child or adult based on their biology alone. Instead, social mechanisms, alone and in interaction with biological mechanisms and random factors likely impact the prognosis and support/treatment needs of autistic people.

The next section discusses social and environmental factors in the *development* or early manifestation of autism. Throughout foetal life, brain development is largely determined by distinct temporal and spatial stages of gene expression and intrinsic neuronal activity. Although it is known that these processes are susceptible to environmental factors, such as malnutrition, alcohol, smoking and drug use, and maternal psychosocial stress, none of these have been specifically linked to autism. After birth, brain development becomes actively refined by interactions with the environment (98). For example, synaptogenesis and plasticity of fronto-parietal, fronto-temporal and fronto-striatal circuits—brain systems underlying higher level social-cognitive and language development—spike between 1 and 3 years (98), which roughly corresponds with the time when social and language-related atypicalities first become apparent in autism. As the newborn turns into an infant and toddler, some of their predispositions interact with increasing exposure to and requirements of the infant/child to engage with more complex and unpredictable environments. Interestingly, whereas genes implicated intellectual disability appear to be predominantly expressed *before* birth, genes linked to autism and neurodevelopmental conditions are often expressed *after* birth [(99), personal communication].

Several theorists have stressed infants’ social visual engagement as early sign of autism. Of note, social visual engagement appears not to be atypical from birth but has been shown to change between 3 and 18 months (100). These early social precursors impact social experiences by altering aspects of the environment that the infant/child acts upon, as well as by modulating the responses from and the interactions with others (101). Recently, Mottron hypothesised that once engagement with non-social aspects in the environment becomes the preferred cognitive style, a bifurcation occurs to the clinically-recognisable “prototypical autism” (32). It suggests a discontinuous process within a specified time-window that results in a categorical outcome. Others regard autistic behaviours as a latent trait comprised of the aggregation of earlier-interacting predispositions (102). Some of these may be specific for autism, such as sensory sensitivities (103), and others domain-general or transdiagnostic (attention, motor coordination) (87, 104). Characteristic continuous autistic traits are thought to emerge as a homeostatic responses or adaptation to the infants’ experiences (105, 106).

Transactional models highlight the role of the dynamics between child and caregivers (and significant others) in the emergence of autism (85). Parents of infants with higher familial likelihood for autism have been shown to adjust to their child in various ways, by offering less social input, or by using more directive or enriched styles to scaffold their child (107). These findings have opened the possibility that changes in the response of the parent could therapeutically influence early developmental processes. In support of this notion, a recent “pre-emptive” intervention trial with infants between the ages of 9–15 months (who had shown early behavioural signs of autism during enrolment) found that video-based parental social-communication training statistically reduced autistic behaviours 24 months afterward (108). These approaches require careful discussion with autistic people as to what outcomes are considered to be positive or desirable, and affirmative of neurodiversity (109).

In sum, these examples highlight that the way the infant/child engages with other people and the world, at each moment, every day, and across development, interacts with critical brain maturation processes. These processes cannot be captured by a reductionist approach that attempts to explain “higher level” phenomena by “lower level” processes in a linear fashion.

## Way forward: Integrating brain, body and the social environment

The precision medicine approach to autism is a framework that was devised by the biomedical community to increase our understanding of the mechanisms underpinning (the development of) autistic subgroups and particular clinical features so to offer tailored support and targeted therapies for core and/or associated features. The ultimate goal is to positively impact the lives of autistic people and their families. Within the ten years I have been working on this approach, empirical findings from our and other studies, insights and criticisms from neurodiversity approaches, and particularly the input of autistic people with lived experience from our AIMS-2-TRIALS “Autism Representatives” have prompted me to revisit some of the assumptions and directions. While this research approach started off with a focus on the individual and search for biological subgroups, the argument made here is that we need to incorporate *both* biological and social mechanisms to better understand the origins of particular autistic features in particular contexts so to make more accurate predictions about a particular person. It broadens the concept of ‘*bio*-markers’ to ‘markers’, defined as an objectively measurable state or characteristic of either a person, environmental condition, or their relationship, in a particular condition or context. This change in focus may lead us to change the term *precision medicine* itself to *precision support* to reflect this broader remit.

Within this framework, it is proposed that new studies require (1) an epistemiological change in how we conduct research, including closer collaboration with autistic people and families to increase the face validity of concepts and methods (110, 111), and explicit acknowledgement of the perspective one adopts; (2) the development of measures that enable repeat (or continuous) assessments of social and biological factors in different conditions and contexts, (3) new models and analytic methods to study (simulate) these interactions, and (4) cross-condition designs to understand which mechanisms are shared (i.e., transdiagnostic) with other neurodevelopmental/neurotypical populations or specific for particular autistic sub-populations. As a consequence, support may entail both interventions for some autistic people or particular features that impact the person’s well-being *and* changes in the environment to create more favourable conditions (including family, school, society at large).

### Is autism a biological entity? When does it matter? For whom?

Even if we are currently still removed from having markers with the strong predictive value needed for clinical utility, it is now the time to work with autistic people and their families to understand what markers are desired and needed, and for what purpose.

Many verbal autistic people emphasise that they recognise each other as being of the same kind—in the absence of a known shared biology. Critically, this recognition and shared identity spans across levels of abilities and support needs, and it is particularly evident in families where family members can substantially differ in their presentation of clinical features. Therefore, it is important to communicate to the autistic community for what purposes subgrouping approaches are expected to be useful in clinical or educational settings, so to avoid potential mis-interpretations and to meaningfully explore acceptance. There are instances where biological characteristics of the individual clearly matter to understand if a given treatment or intervention is likely going to be effective for this person, or to estimate level of side effects. Anecdotally, it appears that for many autistic people efficacy of anti-depressants is lower and side effects can be stronger than for many non-autistic people.

While many researchers have used the term stratification biomarker in a medical context synonymous with sub-division for a particular purpose, in a recent AIMS-2-TRIALS panel discussion (Lisbon, 4th Annual General Meeting, 22 September 2022) it became apparent that some autistic people interpreted it as implying a hierarchy, a better or worse of some subgroups as denoted by social or economic stratification. This would entail unwanted and unintended segregation between autistic people. It is important to understand whether reservations and concerns are to do with such rectifiable miscommunications (by using a different term) or are rooted in more fundamental concerns and disagreements.

Another example of the benefits vs. danger of potential exclusion due to biological subgrouping recently occurred in the wake of scientific advances in Phelan McDermid Syndrome. As said earlier, PMS was originally defined based on chromosomal abnormalities in the 22q1.3 region, and it was later specified that most but not all PMS people have deletion or mutation in SHANK3. Studying specifically participants with SHANK3 haploinsufficiency is important for investigations on the effect of this gene on molecular and cellular processes, but it should not lead to exclusion of people that are part of a community with similar characteristics and needs, and that provides support for each other. To overcome this, a new inclusive classification system was proposed that differentiates between PMS-SHANK3 related and PMS-SHANK3 unrelated (112). Thus, we need to understand how subgroups (including genetically or clinically defined subgroups, such as in the “prototypical autism” proposal) relate to autism and neurodivergence as a whole.

In the AIMS-2-TRIALS biomarker working group with Autism representatives we are currently systematically looking at the acceptability, benefit, ethical and practical concerns of different types of biomarkers for different purposes (“context of use”). It is likely that acceptability and concerns substantially differ between, for example, the use of EEG in predicting epilepsy, (preventative) treatment of hypermobility/pain, cognitive profiles to inform education support, or genetic markers intended for prenatal screening. In fact, in the autistic community, considerable concerns, anxiety and uncertainty related to ethical ramifications of specifically prenatal genetic screening (not pursued in AIMS-2-TRIALS) may have dominated discussions and perceptions of all other types of biomarker research.

Thus, we also need to involve bioethicists and policy makers in these discussions to be aware of and address the ethical and legal ramification for when such markers may become available. This includes fundamental questions, such as legislation around termination and for what purpose, who can take decisions for children and those unable to consent for themselves, or who can access potentially expensive personalised interventions where they are desired.

*New technologies, methodologies and methods:* The conceptual emphasis on the condition and context in which characteristics are measured requires more frequent sampling and in different naturalistic contexts (rather than one-off shot in the experimental lab). Rapid developments of wearables (e.g., actigraphy) and portable, mobile technologies (EEG, fNIRS) promise new ways to assess participants in more naturalistic environments (home, nursery, school), which is expected to increase ecological validity (113). These methodologies likely improve reliability relative to a single snapshot (e.g., MRI scan at one time-point within a longitudinal study) as well as our understanding of context effects and dynamic stability over time (e.g., whether a child consistently shows consistently sustained attention, or varies in different conditions). In our new UKRI funded network, RESPECT4Neurodevelopment, we involve autistic people from the start in the development of the next generation neurotechnologies for infants and children with neurodevelopmental diversity. We also need validated and standardised measures that are comparable across age and ability levels, including children with Intellectual disability, who are often excluded from research (114).

Next, we need new analytic tools to integrate information on biological and social processes. A first step is to create a comprehensive profile or “report card” for each person across different measures acquired. We can then use both data-driven multi-variate approaches (such as clustering) to identify subgroups and theory-driven modelling/simulation approaches to identify additive and interactive mechanisms. Arguably, even data-driven clustering does require some theoretical input (linked to non-trivial variable selection and possible weighting). Different clustering approaches not only face the challenge of robustness but also of finding the subdivisions that are most clinically relevant. Another pivotal problem with Artificial Intelligence algorithms is their focus on classification at the expense of ‘explaining’ their predictions. This has raised the need to get to augment AI with explainable/interpretable AI (XAI) to understand what is inside the black box, and to trace the most predictive factors and mechanisms (115).We also need theory-driven models to study or simulate the dynamics of processes.

*Study designs:* Finally, in order to determine whether any markers and mechanisms are specific to autism (subgroups) or cross diagnostic boundaries, we need cross-condition designs to directly compare autistic participants with participants with other primary neurodevelopmental conditions, such as ADHD and Intellectual disability (116). In our current AIMS-2-TRIALS^1^ and CANDY^2^ biomarker studies, we adopt a life-span approach, with linked studies from infants to adults and characterise each participant in terms of the same transdiagnostic domains, including social, emotional, cognitive, reward, sensory and predictability processing. This includes infant sibling studies (STAARS) where one family member (parent or sibling) is either autistic or has ADHD, which increases likelihood of the infant to develop either neurodevelopmental condition as well as sub-threshold traits, and cross-condition studies, such as the Preschool Brain Imaging and Behaviour Project (PIP), which follows 500 children diagnosed with autism, developmental delay, and/or epilepsy from 3 years of age (and ADHD from 4 years) through to 6 years, multiplex family studies, and experimental medicine studies). We use different study designs as each design has advantages, disadvantages, and systematically affects some participant characteristics (46). For example, PIP children who have received a clinical diagnosis of autism at 3–4 years are likely to have both stronger clinical features and care needs, to comprise a higher rate of co-occurring ID and to come more often from simplex families than autistic or ADHD children identified through infant-sibling designs (which are by definition multiplex). They may also include a higher percentage of Mottron’s “prototypical autism” than LEAP, which includes participants who were diagnosed in adolescence or adulthood. Here, we adopt a more inclusive approach to participant selection (even if rarely truly autism-population representative), which has the advantage that we can directly compare mechanisms and markers between autistic participants that are *a priori* divided by particular characteristics (e.g., the developmental trajectory of “prototypical” vs less prototypical autistic children (7, 32). Hence, the study design needs to be taken into consideration when interpreting results of “subgroups” and replication attempts between study cohorts.

## Conclusion

Over the past decade, biomarker studies aimed at informing precision medicine for autism have substantially influenced the research culture by impacting the design, sample size, quality, method development and methodological rigour. They necessitated and enabled multi-disciplinary collaborations of researchers across different areas of expertise, which more recently includes participatory research with autistic people and families. To date, the majority of studies has focused on identifying biomarkers based on single characteristics (or within the reductionist framework, individual “parts”). This was an important and (certainly from a practical perspective) necessary first step. Findings suggest that while some markers have probabilistic value of clinical utility, so far no characteristic has been identified that can demarcate diagnostic subgroups—as would be required to define autism as a biological entity. In this perspectice article I discussed both conceptual and methodological factors in these findings.

Conceptually, we need to explicitly acknowledge the context/condition in which ‘parts’ are measured, and consider their interactions. This includes the dynamic processes of brain and body over time (with the individuals as a “system”) and dynamic processes of the individual interacting with others in their social environment (as broader social system).

I argued that as a field we are now in a position to develop such an approach. We have set up the infrastructure to conduct multi-disciplinary studies with sample sizes necessary to examine interactions. We have (and are developing) new technologies that allow us to examine participants over time at home, in school, nurseries. And we have changed the research culture to include autistic people and families with lived experience as equal partners in our research to ensure face validity and acceptance of models and methods aimed at increasing autistic well-being.

## Ethics statement

The studies involving human participants were reviewed and approved by local/national ethics committees at each site. Written informed consent to participate in this study was provided by the participants’ legal guardian/next of kin.

## Author contributions

The author confirms being the sole contributor of this work and has approved it for publication.

## Footnotes

1
https://www.aims-2-trials.eu/

2
https://www.candy-project.eu/autism/
